# Promising Natural Medicines for the Treatment of High-Altitude Illness

**DOI:** 10.1089/ham.2022.0139

**Published:** 2023-09-12

**Authors:** Li Li, Lin Lin, Bo Wen, Peng-cheng Zhao, Da-sheng Liu, Guo-ming Pang, Zi-rong Wang, Yong Tan, Cheng Lu

**Affiliations:** ^1^ Institute of Basic Research in Clinical Medicine, China Academy of Chinese Medical Sciences, Beijing, China.; ^2^ School of Life Science, Northwestern Polytechnical University, Xi'an, China.; ^3^ Kaifeng Traditional Chinese Medicine Hospital, Kaifeng, China.; ^4^ Logistics Support Division, National Immigration Administration, Beijing, China.

**Keywords:** high-altitude illness, natural medicines, pharmacological effects, toxicity

## Abstract

Li Li, Lin Lin, Bo Wen, Peng-cheng Zhao, Da-sheng Liu, Guo-ming Pang, Zi-rong Wang, Yong Tan, and Cheng Lu. Promising natural medicines for the treatment of high-altitude illness. *High Alt Med Biol*. 24:175–185, 2023.—High-altitude illness (HAI) is a dangerous disease characterized by oxidative stress, inflammatory damage and hemodynamic changes in the body that can lead to severe damage to the lungs, heart, and brain. Natural medicines are widely known for their multiple active ingredients and pharmacological effects, which may be important in the treatment of HAI. In this review, we outline the specific types of HAI and the underlying pathological mechanisms and summarize the currently documented natural medicines applied in the treatment of acute mountain sickness and high-altitude cerebral edema, high-altitude pulmonary edema, chronic mountain sickness, and high-altitude pulmonary hypertension. Their sources, types, and medicinal sites are summarized, and their active ingredients, pharmacological effects, related mechanisms, and potential toxicity are discussed. In conclusion, natural medicines, as an acceptable complementary and alternative strategy with fewer side effects and more long-term application, can provide a reference for developing more natural antialtitude sickness medicines in the future and have good application prospects in HAI treatment.

## Introduction

High-altitude illness (HAI) is a collective term for a series of maladaptive syndromes that occur at altitudes above 2,500 m, especially acute mountain sickness (AMS) is more common (Li et al., 2018). In recent years, with the rise of tourism and the deepening of economic development, people's demand to enter the highland in a short period has increased, and they are prone to HAI under the influence of multiple factors, such as genetics, altitude of the highland, exercise intensity, and individual constitution (Bartsch and Saltin, 2008; Garrido et al., 2021; Peng et al., 2017). HAI, a common disease in highland areas, can threaten people's physical and mental health and is attracting more attention from researchers.

The treatment of HAI has been a popular topic in modern medicine. Timely administration of appropriate therapeutic measures to HAI patients can reduce morbidity and casualties. Despite the remarkable efficacy of the chemically synthesized drugs currently used to treat HAI, the clinical treatment needs are still not fully met due to various unavoidable problems, such as individual tolerance. The search for new drug treatment strategies remains challenging for researchers to overcome (Murdoch, 2010). Natural medicines have a variety of pharmacological properties and are commonly used to treat a variety of diseases. Traditional Chinese medicine, Tibetan medicine, Ayurveda (India), and other highly distinctive medical systems have a history of herbal therapies for thousands of years, and their treatments for HAI are gaining international attention.

Most natural medicines used to treat HAI are in highland areas, where they are readily available and in good supply and are popular among highlanders. However, little is known about the potential beneficial effects of natural medicines for HAI due to the lack of adequate modern medical research. Therefore, based on the existing clinical and basic studies, in this review, the aim is to summarize the pharmacological properties and possible therapeutic mechanisms of natural medicines with therapeutic effects and make a modest contribution to developing new therapeutic approaches and fully utilizing plant resources.

The types of HAI include AMS, high-altitude cerebral edema (HACE), and high-altitude pulmonary edema (HAPE) in the acute stage. AMS presents atypical symptoms such as headache, vomiting, and discomfort (Berger et al., 2020). AMS can develop into a potentially pathogenic HACE, manifested by ataxia and psychiatric changes, and without timely intervention, the individual may be at serious risk due to cerebral edema (Martí-Carvajal et al., 2012). HAPE is mainly noncardiogenic pulmonary edema induced by hypoxia, manifested by cough, dyspnea, and reduced exercise tolerance (Martí-Carvajal et al., 2012). The risk of acute HAI increases with increasing altitude. The prevalence of AMS is reported to increase by 13% for every 1,000 m increase in altitude above 2,500 m, and the prevalence of HACE and HAPE can be as high as 4.0% (Basnyat and Murdoch, 2003; Meier et al., 2017), which can pose a threat to human health.

The pathological changes in AMS are mainly manifested by an increase in cerebral blood flow and alteration of blood–brain barrier permeability under hypobaric hypoxic conditions. The mechanism of HACE has not been elucidated, but its pathological process is associated with an imbalance of brain self-regulation and the involvement of cytokines (Li et al., 2018). The pathological changes in HAPE include alterations in pulmonary hemodynamics and pulmonary capillary permeability and decreases in alveolar fluid clearance, the inflammatory response, and oxidative stress (Wang et al., 2020a; Zubieta-Calleja and Zubieta-DeUrioste, 2021). Biomarkers related to oxidative stress, such as reactive oxygen species and malondialdehyde; regulatory factors related to blood vessels, such as endothelial nitric oxide synthase, endothelin-1, and vascular endothelial growth factor; and cytokines are all involved in the HAPE pathological process (Lafuente et al., 2016).

People living at high altitudes may develop excess erythrocytosis (EE) and pulmonary hypertension due to long-term chronic hypoxia, resulting in chronic mountain sickness (CMS). The clinical manifestations of CMS include cyanosis, mortar and pestle of the fingers, and dilated veins in the lower extremities, which can progress to cor pulmonale and heart failure in severe cases (Villafuerte and Corante, 2016). It is estimated that 5%–10% of the population is at risk of developing CMS (Leon-Velarde et al., 2005). As a progressive incapacitation syndrome caused by chronic hypoxia, CMS can cause irreversible damage to the brain structure, memory, cognition, and cardiopulmonary function and requires adequate attention (Bao et al., 2019). Other chronic altitude-related diseases such as high-altitude pulmonary hypertension (HAPH) can affect pulmonary artery pressure as well as right and left heart function in high-altitude exposed populations, which is reported to be suspected in 6%–12% of highlanders and the prevalence can be as high as 36% (Lichtblau et al., 2020).

**FIG. 1.**
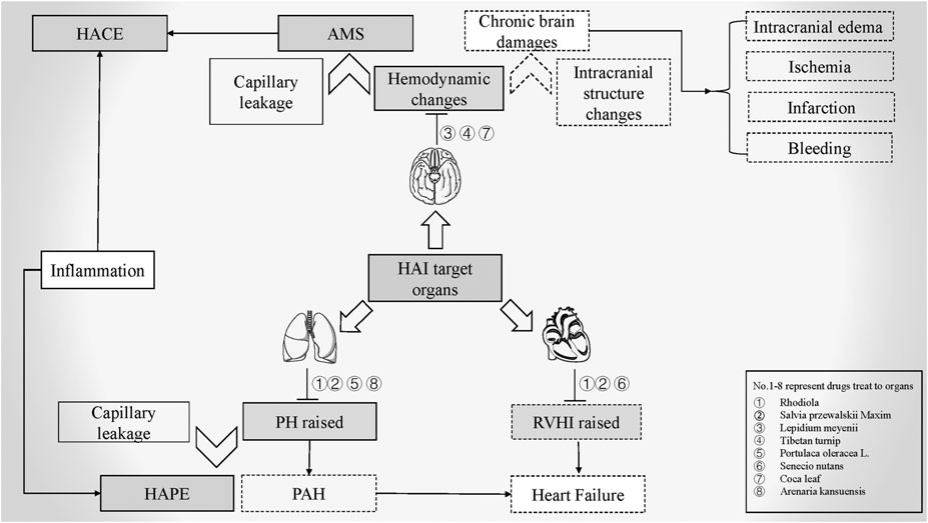
Natural medicines for the treatment of HAI with their target organs. The contents expressed in the *solid line box* are the pathological changes of AMS, and those expressed in the *dotted line box* are the pathological changes of CMS. AMS, acute mountain sickness; CMS, chronic mountain sickness; HACE, high-altitude cerebral edema; HAI, high-altitude illness; HAPE, high-altitude pulmonary edema; PAH, pulmonary artery hypertension; PH, pulmonary hypertension; RVHI, right ventricular hypertrophy index.

Pathological changes in CMS involve the hematological system, lungs, heart, and brain (Bao et al., 2019). The most notable feature of CMS is a compensatory increase in red blood cells (RBCs) due to hypoxia, which leads to EE. In addition, prolonged severe hypoxemia can demonstrate pulmonary arteriole remodeling and right ventricular enlargement due to the vasoconstrictor pulmonary response and the intensity of vascular resistance, which can eventually lead to heart failure (Leon-Velarde et al., 2010; Penaloza and Arias-Stella, 2007). The intracranial structure is changed under long-term hypoxic conditions, which could induce complications such as brain edema, ischemia, infarction, or hemorrhage (Fig. 1).

Timely interventions can reduce the threat of HAI to human health. Acetazolamide and dexamethasone largely improve clinical symptoms, but adverse effects such as paresthesia, fluid retention, and individual intolerance are non-negligible (Gonzalez Garay et al., 2018; Murdoch, 2010). Single herbs and compound herbal preparations, represented by *Rhodiola rosea* and compound Danshen dripping pills, have gradually attracted attention in treating AMS due to their natural origin, various potencies, and few adverse reactions (Li et al., 2020; Yan et al., 2021).

## Search and Study Selection

These natural medicines were obtained by searching PubMed with the subject terms “Altitude sickness” and “Plants, Medicinal.” PubMed-based databases were searched using the following comprehensive terms: ((((((((((((((((((Plants, Medicinal[MeSH Terms]) OR (Medicinal Plant[Title/Abstract])) OR (Plant, Medicinal[Title/Abstract])) OR (Medicinal Plants[Title/Abstract])) OR (Medicinal Herbs[Title/Abstract])) OR (Herb, Medicinal[Title/Abstract])) OR (Medicinal Herb[Title/Abstract])) OR (Herbs, Medicinal[Title/Abstract])) OR (Pharmaceutical Plants[Title/Abstract])) OR (Pharmaceutical Plant[Title/Abstract])) OR (Plant, Pharmaceutical[Title/Abstract])) OR (Plants, Pharmaceutical[Title/Abstract])) OR (Healing Plants[Title/Abstract])) OR (Healing Plant[Title/Abstract])) OR (Plant, Healing[Title/Abstract])) OR (Plants, Healing[Title/Abstract])))) AND ((((((Altitude Sickness[MeSH Terms]) OR (Sickness, Altitude[Title/Abstract])) OR (Altitude Hypoxia[Title/Abstract])) OR (Altitude Hypoxias[Title/Abstract])) OR (Hypoxia, Altitude[Title/Abstract])) OR (Mountain Sickness[Title/Abstract])) OR (Sickness, Mountain[Title/Abstract]).

In addition, a comprehensive search was conducted for AMS, HACE, HAPE, HAPH, CMS, EE, and the natural medicines mentioned above were searched comprehensively for subject terms and free words to ensure systematic and complete searches. The retrieved drugs were obtained after careful review by two researchers, and duplicates and literature with little relevance to the study topic were excluded. The selected natural medicines were further restricted to those with therapeutic rather than preventive effects, and their species, pharmacological properties, and modern medical studies were discussed.

## Natural Medicines Used to Treat HAI

### Acute mountain sickness

#### Rhodiola

*Rhodiola* is a well-known traditional Tibetan natural medicine from the family Crassulaceae with nearly 200 species. Its roots can be used to treat depression, pulmonary fibrosis, metabolic disorders, central nervous system disorders, and AMS (Huang et al., 2020; Liang et al., 2018; Limanaqi et al., 2020; Sangiovanni et al., 2017; Zhang et al., 2016).

In a randomized, single-blind, placebo-controlled trial, after 15 days of administration of *R. rosea* capsules, symptoms such as fatigue, lethargy, chest tightness, palpitations, and dizziness were significantly relieved compared with those in the placebo group for soldiers with signs and symptoms of altitude maladaptation (*p* < 0.05) (Shi et al., 2011). In addition, the combined use of *Rhodiola* and Acetazolamide reversed this increase in hypoxia-inducible factor-1α expression, which was more effective than when they were used separately (Cao et al., 2022).

*Rhodiola crenulata* root extract (RCE) exerts antioxidant effects by impeding oxidative stress biomarkers such as reactive oxygen species and malondialdehyde. Its cardioprotective effects are mediated by restoring the phosphorylation of endothelial nitric oxide synthase in cardiomyocytes and inhibiting arginase-1 under hypoxic conditions, which is related to the phosphatidylinositol 3-kinase (PI3K)/threonine kinase (AKT) signaling pathway (Hsu et al., 2017). In addition, RCE can inhibit the overexpression of endothelin-1 and vascular endothelial growth factor and maintain the normal function of the alveolar–capillary barrier, which is beneficial for alleviating HAPE (Lee et al., 2013).

Salidroside, one of the active ingredients of *R. rosea*, not only resists oxidation and protects the heart but also inhibits the production of inflammatory factors and pulmonary vascular remodeling, potentially benefiting HAPH (Kosanovic et al., 2013). In an experiment in rats with pulmonary arterial hypertension under hypoxia, the levels of RBCs, hemoglobin and hematocrit, right ventricular hypertrophy index, and mean pulmonary artery pressure were reduced by RCE, and vascular smooth muscle thickening and pulmonary vascular remodeling were inhibited (Nan et al., 2018).

**FIG. 2.**
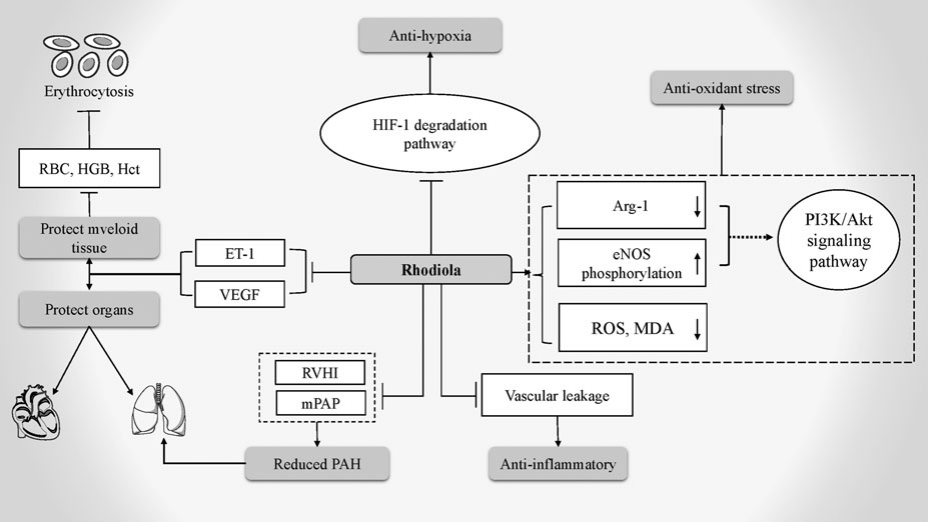
The mechanism of Rhodiola in treating HAI. Arg-1, arginase-1; eNOS, endothelial nitric oxide synthase; ET-1, endothelin-1; Hct, hematocrit; HGB, hemoglobin; HIF-1, hypoxia-inducible factor-1; MDA, malondialdehyde; mPAP, mean pulmonary artery pressure; RBC, red blood cell; ROS, reactive oxygen species; VEGF, vascular endothelial growth factors.

In addition, the anti-inflammatory effect of RCE reduces vascular leakage and repairs acute hypoxia-induced organ damage. Thus, RCE exerts potential therapeutic effects on HAPH and EE (Fig. 2) (Pooja et al., 2009).

#### Tibetan turnip (*Brassica rapa* L.)

Tibetan turnip (*Brassica rapa* L.) is from the genus Brassica in the family Cruciferae, and its roots and leaves are commonly used as a medicine that exerts antihypoxia, antifatigue, analgesic, anti-inflammatory, and neuroprotective effects. People in the Tibetan highland often use it to relieve mild symptoms of CMS. Studies have proven that turnip is composed of phenols and flavonoids, including kaempferol, quercetin, and isorhamnetin, and its root aqueous extract has potent free radical scavenging activity (Chu et al., 2017; Paul et al., 2019).

The turnip also improves the tolerance to hypoxia by increasing the mean corpuscular hemoglobin concentration level to enhance the oxygen-carrying capacity (Chu et al., 2017). By assessing sensorimotor function and microtubule-associated protein 2 in turnip aqueous extract pretreated mice, it was concluded that turnip could improve the repair of neurological damage and prevent neurological deficits. *In vitro*, the turnip aqueous extract can regulate oxidative stress and activate the PI3K/Akt/mTOR signaling pathway to exert neuroprotective effects (Hua et al., 2021). A self-controlled and single-blind human feeding trial showed that the females' peripheral capillary oxygen saturation increased significantly by 6.4% at the end of the hypoxia tolerance test (*p* < 0.05), and the majority of the subjects' hypoxia symptoms improved as well after 7 days of turnip consumption (Chu et al., 2017). These effects benefit highlanders in adapting to the hypoxic environment as they take turnip before engaging in labor in high-altitude areas, indicating that the effects of Tibetan turnip may be acute or subchronic (Chu et al., 2017).

#### *Senecio nutans* SCh. Bip (Asteraceae)

*Senecio* is from the Asteraceae family and is mainly distributed in the Andes, known as “Chachacoma” locally. One species of *Senecio*, called *Senecio nutans* (synonym of *Senecio graveolens*), has the potential to treat cancer and infections, and its leaves are often used to improve symptoms of AMS, including headache, dizziness, vomiting, and fatigue (Echiburu-Chau et al., 2014; Parra et al., 2018). The bioactive ingredients of *S. nutans* include sesquiterpenes, *p*-hydroxyacetophenone, and monoterpenes (Paredes et al., 2016a; Paredes et al., 2016b). *S. nutans* possesses nifedipine-like efficacy that reduces myocardial oxygen consumption and vasodilatory effects (Cifuentes et al., 2016). In addition, its antioxidant and antifatigue effects can help better adapt sojourners to the highland environment (Paredes et al., 2016b). However, current studies on the effectiveness and specific mechanism of action of *S. nutans* in the treatment of AMS are limited and need to be further explored.

#### *Coca* leaf

*Coca* is from the Erythroxylaceae family. Its leaves are often used to treat digestive disorders, depression, throat discomfort, and AMS in South America (Biondich and Joslin, 2016; Biondich and Joslin, 2015; Weil, 1981). The leaf form of *coca* does not produce toxicity or dependence, and *coca* leaves soaked in hot water are still considered the best treatment for nausea, dizziness, and headaches caused by AMS in Andean towns (Weil, 1981).

Cocaine, the primary alkaloid in *coca* leaves, exerts neuroendocrine effects through the hypothalamic–pituitary–adrenal axis, stimulating the sympathetic nervous system and organs such as the brain, heart, and lungs (Biondich and Joslin, 2015; Manetti et al., 2014). Chewing coca leaf containing an average of 0.5% cocaine has been reported to cause a gradual increase in blood levels over most of an hour. The mechanism by which it works to resist AMS may be by blocking the feedback vicious cycle between the stomach or intestines and the central nervous system (Weil, 1981). In addition, *coca* leaves might have an antihypoxia effect that inhibits the excessive increase in RBCs caused by long-term hypoxia (Biondich and Joslin, 2016), which needs to be supported by further experimental data. With the growing demand for natural, less adverse therapeutic agents, *coca* leaves remain a valuable addition to modern treatments, but more testing is needed to ensure the safety and standardization of dosing.

#### Arenaria kansuensis

*Arenaria kansuensis* is a perennial plant in the Caryophyllaceae family that is mainly produced in Qinghai and Tibet of China, and its whole herb is often used to treat AMS, pneumonia, and rheumatoid arthritis (Cui et al., 2018; Cui et al., 2017). *A. kansuensis* contains bioactive ingredients such as steroids, flavonoids, terpenoids, and alkaloids. Its pharmacological effects include antihypoxic, anti-inflammatory, and antipulmonary fibrosis effects (Cui et al., 2021; Cui et al., 2018; Cui et al., 2017). Its antihypoxic activity is related to two active ingredients, pyrocatechol and tricin 7-*O*-β-d-glucopyranoside (Cui et al., 2018). The extract of *A. kansuensis* inhibits proinflammatory cytokines, reduces inflammatory exudates, and improves oxidative stress damage (Cui et al., 2021).

A study showed that treatment with the extract of *A. kansuensis* (100, 300, and 600 mg/kg) in a dose-dependent manner significantly prolonged the survival time of mice exposed to hypoxia and NaNO_2_ poisoning compared with the vehicle control group (Cui et al., 2018). Its antihypoxic and anti-inflammatory activities may play a significant role in the treatment of AMS, and the exact mechanism of action needs to be further investigated.

### High-altitude pulmonary edema

#### *Portulaca oleracea* L

*Portulaca oleracea* L. is a medicinal and edible plant from the Portulacaceae family. As documented in the Chinese Pharmacopoeia, its aerial parts are often used to treat dysentery, carbuncles, and hemorrhagic diseases. Bioactive ingredients, such as flavonoids, alkaloids, terpenoids, and organic acids, can be extracted from *P. oleracea*, exerting antihypoxic, anti-inflammatory, antitumor, and neuroprotective effects (Gu et al., 2022; Zhou et al., 2015).

Histopathology showed that mice in the 100, 200, and 400 mg/kg doses of *P. oleracea* ethanol extract groups had less alveolar flooding and neutrophil infiltration than those in the hypoxic group. The edema score was significantly lower in the high *P. oleracea* ethanol extract group, and both the neutrophil infiltration score and hemorrhage score were significantly lower (*p* < 0.05) (Yue et al., 2015). A dose-dependent antioxidant and anti-inflammatory activity of *P. oleracea* extract against lipopolysaccharide (LPS)-induced acute lung injury in rats has been reported. *P. oleracea* at doses of 100 and 200 mg/kg significantly restored the increase in lung wet/dry ratio compared with the LPS group (*p* < 0.05), with efficacy comparable to that of dexamethasone at 1.5 mg/kg (Baradaran Rahimi et al., 2019). In addition, *P. oleracea* activates the PI3K/Akt and AMP-activated protein kinase signaling pathways in the skeletal muscle of diabetic mice. These two pathways have been implicated in the pathogenesis of HAPE (Lee et al., 2020), and the protective effects and mechanisms of *P. oleracea* by these two pathways need to be further explored.

### Chronic mountain sickness

#### Lepidium meyenii

*Lepidium meyenii* is a Peruvian plant called *maca* and is from the Cruciferous family. It is commonly consumed as a vegetable. Its roots contain fatty acids and polysaccharides and are widely used to treat sexual dysfunction, memory loss, anxiety, nerve damage, tumors, and inflammation (da Silva Leitao Peres et al., 2020; Dording et al., 2008; Sun et al., 2018). *Maca* possesses antioxidant efficacy, and choline in red *maca* can improve neurocognitive function (Gonzales-Arimborgo et al., 2016). Macamides extracted from *maca* exert neuroprotection by promoting the proliferation of neural precursor cells (Cohen-Yeshurun et al., 2013; Wu et al., 2013).

*Maca* was used as a dietary supplement to treat CMS in highlanders (Gonzales-Arimborgo et al., 2016). This study showed that after 12 weeks of using spray-dried *maca* extracts in high-altitude individuals, those in the *maca* group had improved mood, energy, and health compared with the placebo group. Notably, red *maca* was found to significantly reduce CMS scores beginning at week 4 of treatment, and black *maca* began to take effect at week 8 (*p* < 0.05). More interestingly, it was found that black *maca* and a smaller proportion of red *maca* lowered hemoglobin levels at week 4 of administration and targeted only highlanders with abnormally high hemoglobin levels (Gonzales-Arimborgo et al., 2016). However, more studies are needed to confirm the effects and mechanisms of action of different types of *maca* for CMS.

### High-altitude pulmonary hypertension

#### *Salvia przewalskii* Maxim

*Salvia przewalskii* Maxim. (SPM) is from the genus *Salvia* family Labiatae and is mainly produced in northwestern China. It contains bioactive ingredients such as sodium danshensu, rosmarinic acid, and tanshinone (Li et al., 2010; Wang et al., 2020c). The root of SPM is commonly used to treat coronary heart disease, liver disease, renal disease, and stroke, and it has excellent therapeutic potential for HAPH (Skala and Wysokinska, 2005; Wang et al., 2020b; Yang et al., 2017).

*In vivo*, SPM acts as an antioxidant by inhibiting the redox system, and it inhibits fructose metabolism by downregulating the activity of *Khk* and *AldoB* proteins, enabling people to adapt to hypoxia (Wang et al., 2020b). SPM extracts can improve the activities of superoxide dismutase and lactate dehydrogenase in an acute hypoxic environment. Additionally, these extracts can downregulate hypoxia-inducible factor-1α, proliferating cell nuclear antigen, Bcl-2, cyclin-dependent kinase 4, CyclinD1, and P27Kip1; inhibit monocyte chemoattractant protein-1 and nuclear factor-kappaB; and regulate the RhoA-Rho-associated protein kinase signaling pathway, promoting the repair of chronic hypoxia-induced lung injury (Wang et al., 2020c). Overall, the superior antihypoxic activity of SPM makes it beneficial for the treatment of HAPH. More clinical studies are needed to support this efficacy. Relevant natural medicines with therapeutic effects on HAI have been demonstrated in Table 1, while other natural medicines may play a role in the treatment of HAI that are also of interest.

**Table 1.** Classification and Application of Herbs in Treating High-Altitude Illness

Drug	Family	Medicinal parts	Major active ingredients	Clinical application of HAI	References
*Rhodiola*	Crassulaceae	Roots	Flavonoids, Phenylpropanoids, Organic acids	AMS	Limanaqi et al. (2020), Zhang et al. (2016), Huang et al. (2020), Sangiovanni et al. (2017)
Tibetan turnip (*Brassica rapa* L.)	Cruciferae	Roots	Glucosinolates, Isothiocyanates Flavonoids, Polysaccharide	AMS	Paul et al. (2019), Chu et al. (2017)
*Senecio nutans* SCh. Bip	Asteraceae	Leaves	Sesquiterpenes, *p-*hydroxyacetophenones, Benzofurans, Monoterpenes	AMS	Echiburu-Chau et al. (2014), Parra et al. (2018), Paredes et al. (2016a), Paredes et al. (2016b)
Coca leaf	Erythroxylaceae	Leaves	Alkaloid (cocaine)	AMS	Biondich and Joslin (2016), Weil (1981), Biondich and Joslin (2015), Manetti et al. (2014)
*Arenaria kansuensis*	Caryophyllaceae	Whole herb	Steroids, Flavonoids, Terpenoids, Alkaloids	AMS	Cui et al. (2018, 2021, 2017)
*Portulaca oleracea* L.	Portulacaceae	Aerial parts	Flavonoids, Alkaloids, Fatty acids, Polysaccharides, Terpenoids, Sterols	HAPE	Zhou et al. (2015), Gu et al. (2022)
*Lepidium meyenii*	Cruciferae	Roots	*Maca* essential oil, Fatty acid, Polysaccharide	CMS	Gonzales-Arimborgo et al. (2016), da Silva Leitao Peres et al. (2020), Sun et al. (2018)
*Salvia przewalskii* Maxim.	Labiatae	Roots	Tanshinone, Triterpenoids, Phenolic derivatives	HAPH	Li et al. (2010), Yang et al. (2017), Wang et al. (2020b), Skala and Wysokinska (2005)

The table shows eight natural medicines with therapeutic effects, summarizing and summarizing their names, families, medicinal parts, major active ingredients and specific subtypes of HAI, with references. Among them, the most studied botanicals are those related to AMS.

AMS, acute mountain sickness; CMS, chronic mountain sickness; HAI, high-altitude illness; HAPE, high-altitude pulmonary edema; HAPH, high-altitude pulmonary hypertension.

## Promising Natural Medicines for the Treatment of HAI

### *Pleurospermum lindleyanum* (Lipsky) B. Fedtsch

The perennial herb *Pleurospermum lindleyanum*, a member of the Apiaceae family, is widely distributed in western China. Its aerial portions are frequently utilized to treat infections, malignancies, liver illness, coronary heart disease, and hypertension (Zhu et al., 2021). The active components include coumarins, flavonoids, monoterpenes, and aromatic carboxylic acids (Zhu et al., 2021).

*P. Lindleyanum* aqueous extract (5 or 10 g/kg/day) was reported to significantly reduce systolic blood pressure and exert a protective effect on the heart and kidney after 3 weeks of administration (Zhang et al., 2023). In addition, the *P. lindleyanum* aqueous extract increased the plasma nitric oxide level and superoxide dismutase activity in spontaneously hypertensive rats, which exerted antioxidative stress effects (Zhang et al., 2023). These studies indicate that *P. lindleyanum* may play a role in treating HAI. However, its efficacy, mechanism of action, and safety still need to be explored.

### *Xenophyllum poposum* (Phil.) V.A. Funk

*Xenophyllum poposum* (Phil.) V.A. Funk is produced in the Andes and is from the Asteraceae family (Romano et al., 2008). Its aerial parts are often used to treat hypertension, gastrointestinal diseases, rheumatism, and HAI (Cifuentes et al., 2018). The extract of *X. poposum* has antioxidant and cardioprotective effects. It not only exerts a negative inotropic effect but also expands blood vessels by regulating the function of vascular endothelial cells and intracellular calcium concentration, thereby improving cardiac function (Cifuentes et al., 2018; Gonzalez et al., 2012). As *X. poposum* is mainly restricted to local use, its treatment of HAI has only been reported in a limited number of publications. More high-quality evidence is needed to identify the efficacy and mechanism of action of specific HAI.

### *Parastrephia quadrangularis* (Meyen)

*Parastrephia quadrangularis* (Meyen) is a resinous shrub mainly distributed in the central Andes (Cifuentes et al., 2019). It is from the Asteraceae family, known as *Tola-Tola* or *Tolares* locally, and its aerial extracts are often used to treat stomach disorders, infections, and HAI (Ardiles et al., 2018; Di Ciaccio et al., 2018). *P. quadrangularis* contains phenolic compounds, flavonoids, terpenoids, and other bioactive ingredients with antioxidant effects, which can scavenge free radicals and reduce oxidative damage caused by hypoxia.

In normotensive rats receiving *P. quadrangularis* extract (40 mg/kg body weight), blood pressure and heart rate were significantly lower (*p* < 0.01) (Cifuentes et al., 2019). Additionally, it exerts vasodilatory effects through nitric oxide-mediated endothelium-dependent and ion channel-related endothelium-independent mechanisms (Ardiles et al., 2018; Cifuentes et al., 2019). These efficacies may make *P. quadrangularis* play a protective role in treating AMS. Currently, the therapeutic effects of *P. quadrangularis* are limited to few studies, and its clinical application and mechanisms of action have not been elucidated and need to be further explored.

### *Zanthoxylum armatum* DC.

*Zanthoxylum armatum* DC. is a plant in the Rutaceae family, mainly from southern China, India, and Nepal. It contains bioactive ingredients such as polyphenols and flavonoids, and it has an antioxidant effect that scavenges excess free radicals and reduces oxidative stress (Phuyal et al., 2020). The roots, stems, leaves, fruits, bark, and seeds of *Z. armatum* are commonly used to treat HAI, endocrine and metabolic disorders, hypertension, abdominal pain, and headache (Alam et al., 2018; Mushtaq et al., 2019; Phuyal et al., 2020). The methanolic extract of *Z. armatum* has a vasodilatory effect. Its active component *Tambulin* can act on the vascular smooth muscle cells of the coronary artery ring as an endothelium-independent vasodilator (Mushtaq et al., 2019). Its vasodilation and antioxidant effects may be beneficial for alleviating AMS. Current research on *Z. armatum* for the treatment of specific HAI is limited, and more research needs to be invested in *Z. armatum* to fully develop new therapeutic strategies.

## The Toxicity of Natural Medicines Needs Attention

Most natural medicines are safe or less toxic, but some potential toxicity cannot be ignored. It is difficult to evaluate the toxicity of natural medicines in basic research and clinical application because of their diverse ingredients.

The adverse reactions and mortality of SPM lipid-soluble ethanol extracts are dose dependent. When administered by tube feeding, the lethal dose, 50% (LD_50_) of SPM was 2547.8 mg/kg, and adverse reactions were observed at a dose of 1,981 mg/kg, which is a large margin of safety in the clinical application (Li et al., 2010). Coca leaf has cytotoxicity, although it has no significant effect on the stability of the cytogenetic material of coca use (Nersesyan et al., 2013). Cocaine might adversely affect the cardiovascular system, causing cardiac arrhythmia, acute coronary syndrome, and sudden cardiac death (Georgieva et al., 2021). In acute and subacute toxicity tests of the ethanolic extract of *maca*, no significant hepatic and renal toxicities were observed in mice, and its LD_50_ was above 2,000 mg/kg (Yu et al., 2020). However, liver damage and blood pressure abnormalities were reported after volunteers took dried *maca* root powder at 0.6 g/day for 90 days (Valentova et al., 2008).

In summary, the complex compositions and the combined application of herbs have increased the difficulty of assessing the efficacy and safety of herbs to some extent. There are cases of opposite results of toxicity studies in plants.

Natural medicines' different medicinal parts, extracts, and extraction methods need more toxicity studies. Additionally, the administration methods and corresponding detection indicators must be different due to the different physiological structures of humans and animals. In addition, potential clinical adverse events need to be closely monitored to explore strategies to counteract toxicity or adverse reactions to make natural medicines more reliable.

## Conclusions

**FIG. 3.**
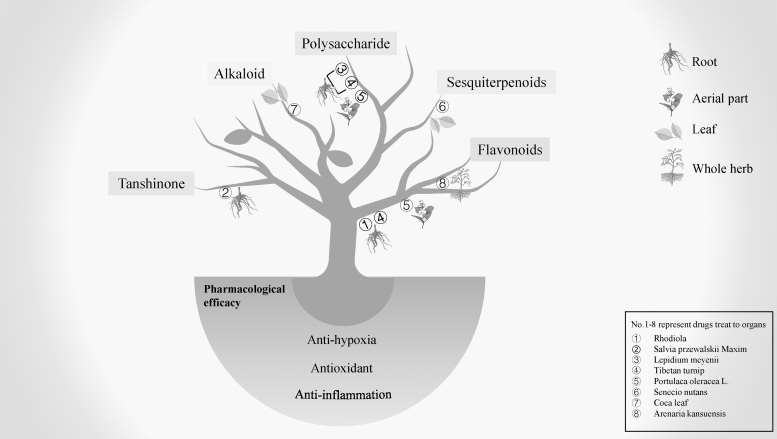
The functions of natural medicines in the treatment of HAI.

In conclusion, natural medicines can be used as alternative therapies for people intolerant to chemically synthesized drugs because of their natural origin, milder effects, and lower toxicity properties (Fig. 3). In addition, plants such as Tibetan turnip could be used as dietary supplements better to acclimatize highland travelers to the hypobaric hypoxic environment and reduce the incidence of HAI. The number of natural medicines known to treat HAI explicitly is limited and lacks relevant reports. In addition, different natural medicines' properties are indicated for specific types of HAI, and their efficacy and safety are primarily dependent on the long-term application experience of users, lacking reliable and high-quality data, which requires more clinical and basic research to elucidate these plants' properties. Overall, potential natural medicines with therapeutic effects on HAI deserve further exploration. There is reason to believe that natural drugs have potential in the treatment of HAI.
